# Editorial: Interactions between bioactive food ingredients and intestinal microbiota, volume II

**DOI:** 10.3389/fmicb.2024.1490884

**Published:** 2024-09-17

**Authors:** Zheng Ruan, Xiaodong Xia, Fengjie Sun

**Affiliations:** ^1^School of Food Science, Nanchang University, Nanchang, China; ^2^School of Food Science and Technology, Dalian Polytechnic University, Dalian, China; ^3^Department of Biological Sciences, School of Science and Technology, Georgia Gwinnett College, Lawrenceville, GA, United States

**Keywords:** bioactive food ingredients, gut health, gut microbiota, microbe-microbe interactions, metabolomics

To date, human health has been greatly benefited from the rapid development of the multiple “omics” techniques (Babu and Snyder, 2023; Karczewski and Snyder, 2018; Dai and Shen, 2022; Mohr et al., 2024; Yurkovich et al., 2024; Hao et al., 2022), in combination with various application strategies, such as the use of dietary nutrients in the regulation of intestinal microbiota (Nayak et al., 2021; Si et al., 2021; Hasin et al., 2017). It is well-known that numerous biological functions, e.g., immune system, energy and intestinal homeostasis of the host, and metabolic activities, involved in human health are regulated by the human gut microbiota, which includes diverse groups of bacteria, fungi, viruses, and protozoa inhabiting the gastrointestinal tract (Fan and Pedersen, 2021; Gebrayel et al., 2022). Therefore, it is extremely important to determine how different diets shape the composition and function of gut microbiome and to explore the associations between diet (nutrition), host, and microbes for the development of precision nutrition and microbiome-based therapies for various medical disorders (Van Hul et al., 2024; Ross et al., 2024; Dmytriv et al., 2024; Jacquier et al., 2024; Li et al., 2024; Pires et al., 2024; Duffuler et al., 2024; Wu et al., 2024; Osawa et al., 2024; Singh et al., 2021; Wang et al., 2019: Han et al., 2021; Ray and Mukherjee, 2021; Deehan et al., 2020). Currently, the molecular mechanisms underlying the interactions among food nutrients, prebiotics, gut microbiota, and host health remain largely unknown.

Considering the rapid advancements in exploring of the associations among food nutrients, gut microbiota, and human health, we emphasize the significance of enhancing global scientific research on the interactions between food nutrient and gut microbiota, and their roles in developing dysbiosis and low-grade inflammation during the intestinal barrier dysfunction and metabolic disorders in hosts and in developing effective dietary treatments for various medical disorders in human.

Building on the success of our Research Topic titled “*Interactions between bioactive food ingredients and intestinal microbiota*” in the journal *Frontiers in Microbiology* (Ruan et al., 2022), we summarize the main results of a total of 15 publications contributed by 115 contributors in Volume II of this Research Topic with the same topics widely explored. Most of the contributions collected in this Research Topic were performed based mainly on animal models, i.e., eight articles on mouse models of a total of six medical disorders, and humans of either healthy subjects or patients of two medical disorders (i.e., colorectal cancer and kidney disease), using various well-established “omics” analyses. The main outcomes of these contributions are summarized below.

First, a total of five contributions were based on healthy human subjects to explore the effects of various substances on the gut microbiome. Vizioli et al. investigated the potential effect of probiotics, yogurt supplemented with *Streptococcus thermophilus, Lactobacillus delbrueckii*, and *Bifidobacterium animalis* subsp. *lactis* strain BB-12, on the gut microbiome and metabolome of a total of 59 healthy children using untargeted metabolomics and shotgun metagenomics analyses. Their results showed that in 10 days, the relative abundances of these probiotics were significantly increased, indicating the impact of probiotics on the bacteria of interest in the gut microbiome. However, the protective gastrointestinal effect of the functional metabolite changes would be verified by longer intervention durations in children at risk for gastrointestinal disorders. Corrêa et al. studied the effects of Moro orange (*Citrus sinensis* L. Osbeck) juice (MOJ) on gut microbiota composition and subsequent changes in the levels of cardiometabolic biomarkers in a total of 12 overweight women, demonstrating the importance of orange juice intake duration, as observed in the evident variations in the beneficial changes (e.g., blood pressure improvement) between 2- and 4-week interval of MOJ intake. This study provided strong experimental evidence to support that changes in specific operational taxonomic units (OTUs) of the gut microbiota in response to MOJ intake were associated with significant improvements in some cardiometabolic biomarkers and short-chain fatty acid (SCFA) levels in overweight women with insulin resistance. Gu et al. explored the potential effects of citrus pectin-type polysaccharides, including (1) the pectin polysaccharide (PEC), which was the partially hydrolyzed pectin (PPH), and (2) the pectin oligosaccharide (POS), on the improvement of aging-associated dysbiosis and the levels of SCFAs of the gut microbiota using five healthy elderly volunteers (70–75 years) and five younger adults (30–35 years). The main results showed that these pectins boosted various bacterial groups differently from the reference prebiotic substrate (inulin), and the *in vitro* modulating effects of pectins on elderly gut microbiota revealed significant potential of using pectins to improve age-related dysbiosis. The authors indicated that further human intervention studies were necessary to verify the potential effects of pectins observed in this study. Rawi et al. identified the putative primary degraders (i.e., gum-fermenting bacteria) of commercial acacia (*Acacia senegal*) gum, which was composed of arabinogalactan branched polysaccharide and was marketed as a functional dietary fiber to improve overall human gut health, in the gut ecosystem of three healthy human subjects based on enrichment culture fermentation in an anaerobic chamber for 144 h. Based on the *16S RNA* sequencing, a total of five bacterial strains were found to be gum-fermenting bacteria and matched to butyrate-producing *Escherichia fergusonii*, ATCC 35469. This study confirmed the use of acacia gum as a potential prebiotic and an alternative approach for mediating gut illness. Lan et al. screened strains of *Bifidobacterium animalis* subsp. *lactis* with differential oligosaccharide metabolism to subsequently perform genome-wide resequencing and real time quantitative PCR (RT-qPCR) analyses in mothers and their infants. The authors further explored the mechanism underlying the differences in *B. animalis* subsp. *lactis* oligosaccharide metabolism, revealing that the variations in the gene transcription levels led to intraspecies differences in the ability of the strains to metabolize oligosaccharides even when they belonged to the same subspecies, providing strong experimental evidence to support the utilization of *B. animalis* subsp. *lactis* strains as probiotics and the development of synbiotic products.

Second, two publications were based on patients of two diseases, colorectal cancer and kidney disease. Kim et al. assessed the effects of a modified microbiota-accessible carbohydrate (mMAC) (high-fiber) diet on gut microbiota composition and clinical symptoms in two groups of a total of 40 colon cancer patients who underwent surgical resection, those who received adjuvant chemotherapy and those who did not. The main results included the distinct differences in gut microbial composition after the mMAC diet in both the chemotherapy and non-chemotheraphy groups, providing valuable insights into the potential benefits of the mMAC diet, specifically its impact on the gut microbiome and clinical symptoms in postoperative colorectal cancer patients. Lazarevic et al. explored the alternations in gut barrier, composed of gut microbiota and playing pivotal roles in chronic kidney disease (CKD) progression and nutritional status, in a total of 22 hemodialyzed (HD) patients and 11 non-HD (NHD) CKD patients. The main results included that compared to healthy group, HD patients exhibited significant alterations in fecal microbiota composition, a higher systemic inflammation, and a modification in plasma levels of appetite mediators. This study underscored the multifaceted interplay among gut microbiota, physiological markers, and kidney function. It was noted that further investigations in larger cohorts were necessary to verify the findings revealed in this study.

Third, a total of three articles were based on mouse models of colitis. Xia et al. investigated the influence of *Litsea cubeba* essential oil (LCEO) on lipopolysaccharides (LPS)-induced mouse intestinal inflammation model and associated changes in the gut microbiota, i.e., the therapeutic potential of LCEO for gut health, with particular emphasis on its gut protective properties, as well as the anti-inflammatory properties and modulation of the gut microbiome. This study identified LCEO as a promising natural compound for ameliorating diarrhea and intestinal inflammation, highlighting the need to further explore the complex interplay among the host, gut microbiome, and natural products in the context of inflammatory diseases. Wu et al. examined the effects of an intervention with fructooligosaccharides (FOS), *Saccharomyces boulardii*, and their combination in a mouse model of colitis and to explore the mechanisms underlying these effects. The results revealed enhanced anti-inflammatory effects of the combined administration of FOS and *S. boulardii* in treating colitis and colitis-induced intestinal dysbiosis, in comparison to the application of FOS alone. In particular, the combination significantly increases the abundance of beneficial bacteria such as *lactobacilli* and *Bifidobacteria* and effectively regulated the gut microbiota composition, providing a scientific rationale for the prevention and treatment of colitis using a combination of FOS and *S. boulardii* and for the development of nutraceutical preparations containing both FOS and *S. boulardii*. Zou et al. applied network pharmacology to mine and verify the single active ingredient, puerarin, in *Radis puerariae*. The authors found that puerarin was a potential ingredient that could improve the crypt deformation and inflammatory infiltration in mice, as observed in the decreased levels of various inflammation related factors, while the results of correlation network and metabolic function prediction analysis of the microbiota revealed a tightly connected network widely involved in carbohydrate metabolism and amino acid metabolism. This study revealed high therapeutic effect of puerarin on ulcerative colitis, which was partially achieved by restoring the composition and abundance of gut microbiota and their metabolism.

Lastly, a total of five contributions were based on mouse models of five different medical disorders. Tian et al. investigated the effect of ursolic acid on obesity depending on the regulation of gut microbiota and metabolism based on the mouse model of obesity established with a high-fat diet using intestinal microbiome and metabolomics analyses. The results revealed that the roles of ursolic acid in the anti-obesity process depended in part on alterations in the gut microbiota and metabolism, highlighting the potential therapeutic effect of ursolic acid on the improvement of diet-induced obesity in humans. Zang et al. explored the mechanism of red quinoa polysaccharide (RQP), which was a complex polysaccharide containing more glucose, galactose and acarbose, in alleviating type 2 diabetes (T2D) through both *in vivo* and *in vitro* experiments using the mouse model of T2D induced by high-fat diet. The results showed strong anti-diabetic effects of RQP on T2D and transformed intestinal microbiota composition in diabetic mice, revealing that RQP could inhibit the development of diabetes by correcting the imbalance of intestinal microbiota. Hsiao et al. investigated the use of okra (containing a viscous substance rich in water-soluble material, including fibers, pectin, proteoglycans, gum, and polysaccharides) polysaccharides by microorganisms and their potential to improve microbiota by assessing the regulation of microcapsules prepared from okra polysaccharides with or without *Lactiplantibacilus plantarum* encapsulation on intestinal microbiota through 16S rRNA metagenomic analysis and SCFAs in the mouse model of Alzheimer's disease (AD). Interestingly, the authors found that both microcapsules prepared from okra polysaccharides either with or without *L. plantarum* encapsulation improved intestinal microbiota by elevating *Lactobacillus* levels in AD mice. Feng et al. explore the effects of alcohol intake at different concentrations on gouty arthritis based on the gut microbiota in the mouse model of acute gouty arthritis established by injection of monosodium urate crystals. Based on various morphological and biochemical factors, it was concluded that alcohol of high concentration altered the gut microbiota structure in gouty mice, possibly exacerbating gouty symptoms by enhancing pro-inflammatory pathways. Hua et al. investigated the mechanisms underlying the attenuation effect of hemp seed (HS) and its water-ethanol extract on mice with loperamide-induced constipation. The gut microbiome studies showed that the structure and abundance of intestinal microbiome were altered, as observed in the changed relatively abundances of *Odoribacter, Bacteroide, Lactobacillus*, and *Prevotella*. This study revealed the potential of HS to stimulate the proliferation of beneficial gut microbes and promote intestinal motility, thereby improving gut health and relieving symptoms of constipation.

In summary, this Research Topic of contributions highlights recent progress in the relevant fields of human health discussed in this Research Topic and suggests future research directions with potential avenues for scholars to explore. Given the rapid development of techniques for exploring the interactions between bioactive food ingredients and intestinal microbiota, it is expected that significant achievements related to human health will soon emerge from the topics covered in this Research Topic.
